# Blood-Mediated Gut–Brain Axis in Parkinson’s Disease: Focus on α-Synuclein Transport and Microbiota Dysbiosis-Induced Inflammation

**DOI:** 10.3390/cells15181718

**Published:** 2026-09-21

**Authors:** Chunjie Xu, Wei Li, Xiaonan Ma, Luyu Han, Guangxu Cui, Jingtong Zhao, Xin Wang, Yingjun Guan

**Affiliations:** 1 Department of Histology and Embryology, School of Basic Medical Sciences, Shandong Second Medical University, Weifang 261053, China; xu1261781793@163.com (C.X.); liwei9549@sdsmu.edu.cn (W.L.); 18855241172@163.com (X.M.); 18535582002@163.com (L.H.); 2 Neurologic Disorders and Regenerative Repair Laboratory, Shandong Second Medical University, Weifang 261053, China; 17615900025@163.com (G.C.); 13864658388@163.com (J.Z.); 3 Department of Neurosurgery, Brigham and Women’s Hospital, Harvard Medical School, Boston, MA 02115, USA

**Keywords:** blood, gut–brain axis, α-synuclein, inflammation, microbiota dysbiosis, Parkinson’s disease

## Abstract

**Highlights:**

**What are the main findings?**

- Reviews the pathological transport of α-synuclein across the gut barrier and blood–brain barrier.
- Summarizes how gut microbiota dysbiosis may affect brain pathology via blood-mediated inflammation.

**What are the implications of the main findings?**

- Highlights the potential role of blood in mediating gut–brain crosstalk in Parkinson’s disease.
- Identifies potential biomarkers and therapeutic strategies targeting the gut–blood–brain axis.

**Abstract:**

Parkinson’s disease (PD) is a progressive neurodegenerative disorder characterized by the loss of dopaminergic neurons and the deposition of Lewy bodies (LBs). The gut–brain axis has emerged as a potential route for the bidirectional dissemination of PD pathology, in which the enteric and central nervous systems may contribute to the spread of misfolded α-synuclein (α-Syn) and associated neuroinflammatory responses. However, neural pathways alone may not fully account for the widespread distribution of PD pathology across the brain and gut. As another major conduit connecting the gut and the brain, the peripheral circulation may provide an additional route for PD-related pathological processes. Nevertheless, how blood circulation contributes to the development and dissemination of pathology along the gut–brain axis remains underexplored. Accordingly, this narrative review examines peripheral blood as a potential additional route for pathological communication along the gut–brain axis, focusing on two potential mechanisms involving the transport of pathological α-Syn aggregates between the gut and the brain and the circulation of inflammatory signals associated with gut microbiota dysbiosis. Furthermore, to highlight the translational relevance of the gut–blood–brain axis, we briefly summarize recent advances in related blood-derived biomarkers and therapeutic strategies targeting these pathways.

## 1. Introduction

Parkinson’s disease (PD), the second most prevalent neurodegenerative disorder globally, is characterized by the degeneration of dopaminergic neurons in the substantia nigra and the formation of Lewy bodies (LBs) in both central and peripheral tissues [1]. Under pathological conditions, α-synuclein (α-Syn) misfolds and aggregates to form the major component of Lewy bodies (LBs) [1]. Pathological α-Syn deposition has been detected throughout the gastrointestinal tract, and constipation is a common non-motor manifestation of PD that may occur during the prodromal phase, before the onset of motor symptoms [2,3]. These pathological and clinical manifestations in the gut have driven extensive investigation of the gut–brain axis in PD.

The “gut–brain axis” is a complex communication network involving chemical, neuronal and immunological signaling between the gastrointestinal tract and the brain. Multiple studies have implicated this bidirectional network in PD pathogenesis. For example, bilateral injections of an α-Syn-encoding adeno-associated virus into the substantia nigra of rats induced significant changes in both the ENS and gut microbiota, further highlighting the crucial involvement of the gut–brain axis in disease pathogenesis [4]. PD is associated with marked gut dysbiosis, and the ENS exhibits α-Syn aggregation, gut inflammation, and deficits in dopaminergic signaling [5]. In addition, alterations in gut microbial composition and activity have been associated with α-Syn aggregation, increased intestinal permeability, neuroinflammation, oxidative stress, and altered neurotransmitter production [6], suggesting that pathological changes in the gut may influence brain homeostasis.

Neural communication plays a vital role in the gut–brain axis, especially in the bottom-up transmission of synucleinopathy. According to Braak’s hypothesis, this pathology begins in the ENS and spreads through the dorsal motor nucleus of the vagus nerve into the lower brainstem and ultimately reaches the midbrain and the cerebral cortex [7], which is consistent with the “gut-first” hypothesis [6,8]. However, the pattern of Lewy pathology proposed in Braak’s staging scheme has not been consistently observed in all patients [9]. Clinical studies indicate that up to 47% of cases do not follow the ascending progression of Lewy pathology [10,11]. Injection of α-Syn preformed fibrils into the gastrointestinal tract of mice induced progressive brain α-Syn pathology that was prevented by truncal vagotomy, supporting the possibility of vagus-mediated gut-to-brain propagation [12]. In contrast, another study found that enteric α-Syn pathology failed to induce sustained CNS pathology in rodents or nonhuman primates, suggesting that persistent gut-to-brain propagation may be uncommon [13]. Neuropathological studies have also shown that many patients with typical PD have no detectable α-Syn accumulation in the dorsal motor nucleus of the vagus despite severe degeneration in the substantia nigra and spinal cord [14]. Although neural pathways may contribute to the spread of pathology from the gut to the brain, these inconsistent findings suggest that they do not fully account for the observed heterogeneity of PD pathology. Other routes of gut–brain communication therefore warrant further investigation.

As PD is increasingly recognized as a systemic disorder, peripheral blood may serve as an additional medium for communication among different tissues (including the gut and brain) beyond the nervous system. However, whether and how the peripheral blood circulation contributes to the propagation of PD pathology along the gut–brain axis remains underexplored. Growing evidence indicates that aberrant α-Syn aggregates and α-Syn-containing extracellular vesicles are detectable in the peripheral blood of patients with PD [15]. In addition to the wide distribution of α-Syn, PD is increasingly recognized as a disorder involving systemic inflammatory responses. Compromised blood–brain barrier (BBB) integrity could facilitate the entry of circulating inflammatory mediators into the brain. Gut-derived microbial metabolites and endotoxins may also enter the systemic circulation and induce inflammatory responses, potentially amplifying peripheral-to-central pathological cascades [16]. Therefore, we provide a narrative overview of the potential contribution of blood circulation to the gut–brain axis, focusing on the transport of α-Syn aggregates and microbiota dysbiosis-induced inflammation. We further discuss the potential diagnostic and therapeutic implications of these processes in PD and related neurodegenerative diseases to highlight the role of the gut–blood–brain axis.

## 2. Search Strategy

The PubMed/MEDLINE database was used for the research, with only articles in English language, using the following terms alone or in combination: “Parkinson’s disease”, “α-synuclein”, “gut-brain axis”, “blood”, “enteric nervous system”, “gut microbiota”, “gut dysbiosis”, “intestinal inflammation”, “systemic inflammation”, “neuroinflammation”, “gut barrier”, “blood-brain barrier”, “extracellular vesicles”, “red blood cells”, “peripheral blood mononuclear cells”, “microbial metabolites”, “lipopolysaccharide”, “short-chain fatty acids”, “bile acids”, “tryptophan metabolites”, “trimethylamine N-oxide”, and “biomarkers”. Studies were included in this narrative review if they addressed communication among the gut, peripheral circulation, and brain in PD or provided relevant mechanistic evidence regarding α-Syn transport or microbiota dysbiosis-induced inflammation. The literature was searched from inception to 2026, including original research articles, review, meta-analysis, systematic review, viewpoint and case reports. Titles and abstracts were screened for relevance, followed by full-text assessment of potentially eligible articles.

## 3. Pathological α-Syn Transport Between the Gut and Brain via Blood

### 3.1. Distribution of α-Syn in the Gut, Brain and Blood

α-Syn is present in neuronal and non-neuronal cell types, peripheral organs, and body fluids, including the brain, skin, gut, salivary glands, blood, and cerebrospinal fluid (CSF) [3,17,18]. In the normal brain, α-Syn is detected in close proximity to presynaptic terminals or within the glial cytoplasm at various sites of the CNS, except in the primary visual and cerebellar cortex [19]. In contrast, Lewy pathology has been identified in the dorsal motor nucleus of the vagus, hypothalamus, substantia nigra pars compacta, temporal mesocortex, and premotor areas in Braak’s staging scheme [7]. In the gut, the ENS is the main source of gut-derived α-Syn aggregates. Almost 40 years ago, researchers reported the presence of LBs and neurites in the gut, which were subsequently confirmed in the gastrointestinal tract of patients with PD through autopsy studies [20,21]. Lewy pathology is distributed in both the myenteric and submucosal plexuses from the upper esophagus to the rectum [3]. Detection of pathological α-Syn in gut biopsy tissue provides a potential diagnostic biomarker of PD. In one study, RT-QuIC analysis of duodenal biopsies yielded a sensitivity of 95.7% and a specificity of 100% for PD [22]. α-Syn is also expressed in enteroendocrine cells (EECs) of the gut mucosa, which may represent a non-neuronal source of fibrillar α-Syn in the gut [23,24]. In the brain, pathological α-Syn species can impair mitochondrial function, increase oxidative stress, activate microglia, and promote neuronal injury [25,26]. In the gastrointestinal tract, α-Syn-related pathology has been associated with dysfunction or loss of enteric neurons, including dopaminergic neurons, as well as with enteric gliosis and local inflammatory responses [27,28].

It is increasingly recognized that multiple α-Syn species are present at relatively high levels in whole blood. The detection of pathological α-Syn conformers in plasma, serum, red blood cells (RBCs), and neuron-derived EVs from blood samples has the potential to serve as a blood biomarker of PD [29,30]. Using an α-Syn seed amplification assay, these alterations have been detected in peripheral blood up to 10 years before the clinical diagnosis of PD [31]. Notably, studies indicate that over 99% of α-Syn is found within RBCs, while plasma, mononuclear cells and platelets account for much lower levels [32]. As a result, measurements of α-Syn levels in plasma show inconsistent trends [33,34]. Numerous studies have found that RBCs from patients with PD show a significant increase in total, monomeric, oligomeric, aggregated and phosphorylated α-Syn species [35,36,37]. In the gut, blood and brain tissue, total α-Syn, phosphorylated α-Syn, α-Syn aggregates and EV-associated α-Syn all show similar yet variable trends, indicating that pathological α-Syn in the blood may effectively reflect pathological processes in both the intestinal tract and brain (Table 1).

### 3.2. Blood–Brain Barrier and Gut Barrier Dysfunction in PD

The BBB is a highly selective semipermeable membrane barrier that separates circulating blood from the brain and the extracellular fluid of the central nervous system (CNS). Increasing evidence indicates that the BBB is altered in PD. Exposure to α-Syn oligomers activates astrocytes, enhancing the release of vascular endothelial growth factor A (VEGFA) and nitric oxide (NO), both of which impair BBB integrity [74]. Postmortem analyses of striatal tissue obtained from patients with PD reveal extravasation of serum proteins, including albumin and fibrinogen [75,76]. These findings indicate increased BBB permeability in PD patients, which likely affects neurological function. Monocyte infiltration into the substantia nigra and striatum has also been associated with neuroinflammation and BBB disruption in PD [75,76]. Additionally, albumin leakage into CSF has been detected in both healthy controls and PD patients, signifying BBB impairment [75].

The gut microbiota regulates immune responses and maintains the integrity of the BBB. Research shows that PD patients have a higher prevalence of *Helicobacter suis* infection, which activates microglia and leads to cognitive impairment. Although the BBB remains intact, *Helicobacter suis* compromises gastrointestinal integrity, triggering peripheral inflammation that affects brain homeostasis through the blood-CSF barrier [77]. The human gastrointestinal tract is a key source of peripheral dopamine, with bacterial species influencing neuroimmune responses through the release of cytokines and neurotransmitters. In patients with PD and in rodent models, dopamine concentrations and receptor expression may be altered in both peripheral tissues and the CNS [78]. As PD progresses, gut microbiota interacts with microglia, regulating their maturation and function in inflammatory responses. Microglia release pro-inflammatory cytokines and chemokines including TNF-α, IFN-β, IFN-γ, and IL-1β, which compromise BBB integrity and recruit lymphocytes to sites of neuronal injury [78].

The gut barrier, including the mucus layer, epithelial barrier, and gut–vascular barrier (GVB), maintains intestinal homeostasis and helps coordinate brain physiology through the gut–brain axis [79]. Both patients with PD and animal models exhibit alterations in gut-barrier integrity. Studies have reported increased intestinal permeability in PD; for example, colonic biopsies show reduced occludin expression and altered subcellular distributions of occludin and ZO-1 [80,81]. In an α-Syn transgenic mouse model, intestinal inflammation and epithelial barrier disruption promoted enteric α-Syn accumulation and subsequently exacerbated age-dependent brain pathology [82]. In a low-dose oral rotenone mouse model, stress-induced intestinal barrier damage, accompanied by disruption of tight junction proteins (ZO-1, occludin, and claudin-1), α-Syn accumulation, and endotoxemia, drives a pro-inflammatory milieu that exacerbates PD pathology [83].

GVB, also termed the gut-blood barrier, is located between the gut and the bloodstream and resembles the BBB in its high selectivity for molecular transport and in its regulation of microbiota-derived substances entering the circulation [84]. Beyond the gut mucosal layer and its epithelial barrier, GVB comprises endothelial cells (ECs) connected by tight junctions and adherens junctions, as well as enteric glial cells, pericytes, and fibroblasts, which serve as the ultimate checkpoint to maintain gut-blood homeostasis [84,85]. GVB disruption due to an inflammatory insult can induce closure of the choroid plexus vascular barrier in mice [86]. Increasing evidence suggests that gut microbiota dysbiosis is associated with multiple extraintestinal pathologies and may induce GVB leakage at early disease stages [87]. Damage to the GVB may increase circulating levels of microbes, cytokines, and other inflammatory mediators, thereby promoting low-grade systemic inflammation that can affect distal organs, including the brain [88]. The canonical Wnt/β-catenin signaling pathway plays a fundamental role in maintaining GVB integrity, and increasing evidence indicates aberrant Wnt/β-catenin signaling in PD [84,89,90].

### 3.3. α-Syn Transport Across the BBB and Gut Barrier

**Figure 1:**
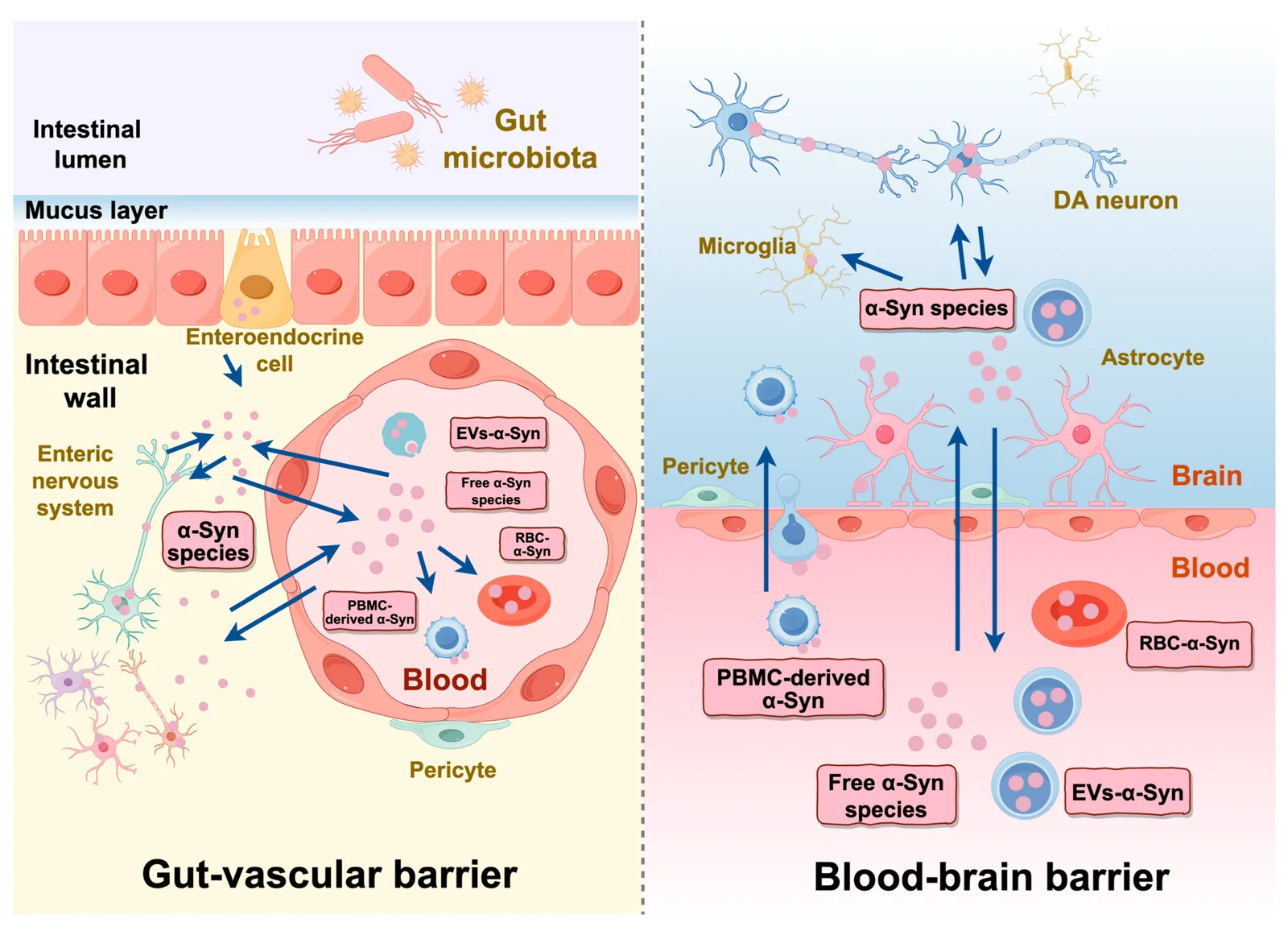
Blood-associated transport of α-Syn species across the gut barrier and BBB in PD. Intestinal inflammation and immune dysfunction may promote α-Syn aggregation and release from enteroendocrine cells, macrophages, enteric neurons, and enteric glial cells. Under conditions of gut–vascular barrier dysfunction, some of these α-Syn species may gain access to the circulation. Conversely, circulating free or vesicle-associated α-Syn species may enter intestinal tissues. BBB dysfunction may also facilitate the exchange of α-Syn species between the circulation and brain. These proposed pathways may provide an additional route of gut–brain communication, although their directionality and causal contribution to human PD remain incompletely established. This figure was created by Figdraw.

Dysfunction of the BBB and gut barrier allows pathological α-Syn transport between the brain and gut via the bloodstream (Figure 1). Elevated levels of α-Syn in serum or plasma and mature RBCs from PD patients suggest bidirectional transport of α-Syn between the brain and blood. Previous studies have shown that radioactively labeled α-Syn can cross the BBB in both the brain-to-blood and blood-to-brain directions at comparable rates [91]. α-Syn fibrils are capable of entering the CNS after oral or intravenous administration, subsequently promoting neuropathological changes [92]. The circulation hypothesis posits that pathological α-Syn originates in the brain as a result of BBB disruption and crosstalk between blood and CSF, and it subsequently influences disease progression via the autonomic nervous system [93]. Low-density lipoprotein receptor-related protein 1 (LRP-1), a bidirectional transporter, plays a crucial role in mediating the transport of α-Syn across the BBB. This mechanism allows α-Syn to cross the BBB bidirectionally, underscoring the complex interactions between peripheral and central nervous system pathologies [93]. When blood-associated α-Syn aggregates are transported across the BBB, they may increase the cerebral burden of pathological α-Syn and seed further misfolding and prion-like propagation of endogenous α-Syn. Although elevated α-Syn aggregates may generate widespread vascular, immune, and proteostatic stress within the central nervous system, dopaminergic neurons in the substantia nigra are particularly susceptible to additional stress caused by pathological α-Syn [94]. For example, their autonomous pacemaking and associated Ca^2+^ influx generate a persistent basal level of mitochondrial oxidant stress, which can be further exacerbated by intracellular α-Syn aggregation [95,96].

Pathological α-Syn species can alter tight junction protein expression and the release of vasoactive or inflammatory mediators from endothelial cells, thereby affecting BBB maintenance [97]. A damaged BBB also allows α-Syn to infiltrate the brain, contributing to neurodegeneration, particularly under conditions of LPS-induced BBB disruption. This disruption leads to increased permeability of the BBB, facilitating the passage of potentially harmful substances, including α-Syn aggregates [91]. In an in vitro BBB model system, both α-Syn monomers and oligomers were internalized by primary brain ECs, while the transport of oligomeric α-Syn was more restricted than that of monomers. Furthermore, RBCs have been observed to release EVs containing α-Syn capable of crossing the BBB, and active transcytosis is suggested as the primary transport mechanism, which can be inhibited at low temperatures or by endocytosis inhibitors [98,99]. This transport is also enhanced during inflammation due to LPS-induced BBB permeability [99]. Recent work further demonstrates that bone marrow-derived erythrocytic α-Syn enters the CNS and preferentially accumulates in microglia, contributing to dopaminergic neurodegeneration, while blood–brain barrier integrity critically regulates this peripheral-to-central pathological transmission [100].

In addition to enteric and extrinsically innervating neurons, there are a number of cells that natively produce α-Syn within the GI tract, such as epithelial EECs [101]. The intestinal tract receives abundant blood flow. Pathological conditions may facilitate the release of α-Syn from enteric and other intestinal cells and its subsequent passage across a compromised GVB. Studies have detected increased levels of α-Syn-containing neuron-derived EVs in the blood of patients with PD, providing evidence that EVs may serve as transport vehicles facilitating communication through systemic circulation [102]. Moreover, EVs derived from erythrocytes in human blood contain α-Syn, with RBCs being the primary source of α-Syn in the bloodstream [32,99]. Significant accumulations of RBC-EVs have been observed in the cecum and colon, and these accumulations were significantly influenced by butyrate and α-Syn genotypes and directly affected the gut microbiome [103]. In addition, RBC-derived EVs containing aggregated α-Syn can enter monocytes through receptor-mediated endocytosis [104]; these monocytes may potentially migrate to the intestine during inflammation.

### 3.4. Role of Peripheral Blood Mononuclear Cells in α-Syn Transport

Peripheral blood mononuclear cells (PBMCs), including T cells, B cells, and monocytes, are important components of the peripheral immune system and may contribute to the pathogenesis of PD. An increasing number of studies have shown that gut dysbiosis may modulate PBMCs inflammatory activity and subsequently promote PD progression [105]. Research has examined how α-Syn moves from the gut to the CNS and the role of CD4+ T cells in this process, highlighting that lymphocyte-activation gene 3 (LAG3) regulates CD4+ T cell proliferation and function while specifically binding to pathological α-Syn [106]. Several studies have shown that activated CD4+ T cells can cross the BBB from the periphery and can be detected in brain tissue samples, where they contribute to central inflammatory responses and infiltrate the substantia nigra in PD patients, a process closely linked to the formation of LBs [107]. In addition, recent studies have demonstrated elevated CD3+ T cell counts in intestinal biopsies from PD patients with constipation and in PD mouse models exhibiting prodromal constipation, as well as increased T cell populations in the plasma and brain of PD patients, although their exact source remains unclear [108].

## 4. Influence of Gut Microbiota Dysbiosis on the Brain Through Blood-Mediated Systemic Inflammation

Under physiological conditions, α-Syn is present in the gut without overt pathological aggregation. Changes in the intestinal microenvironment, particularly those associated with gut microbiota dysbiosis and inflammation, may promote α-Syn misfolding and aggregation [109]. Disruption of the intestinal and gut–vascular barriers may further create conditions permissive for pathological α-Syn species to gain access to the circulation, although direct evidence demonstrating the entry of gut-derived α-Syn into the bloodstream remains limited. Conversely, aggregated α-Syn may further promote local inflammatory responses in the gut, potentially creating a self-reinforcing cycle between α-Syn aggregation and intestinal inflammation [110]. The effects of gut dysbiosis are not restricted to intestinal α-Syn pathology. Microbial products and inflammatory mediators may enter the circulation, alter the systemic inflammatory milieu, and subsequently affect BBB integrity, neuroinflammation, and central nervous system homeostasis.

### 4.1. Characteristics of Gut Microbiota Dysbiosis in PD

The gut microbiota and its metabolites participate in diverse processes, including regulation of gastrointestinal physiology, maintenance of gut-barrier integrity, support of key metabolic pathways, and maintenance of immune homeostasis [111]. Dysbiosis may alter cytokine and metabolite profiles and may thereby influence gut-barrier and BBB permeability, neuroinflammation, and neurodegenerative processes [112].

In patients with PD, gut microbiota dysbiosis has been consistently observed, with notable reductions in beneficial bacteria. For instance, *Lactobacillaceae*, *Prevotellaceae*, *Bifidobacterium* and *Lachnospiraceae* were decreased [113,114], while *Pasteurellaceae* and *Enterobacteriaceae* were increased [115]. *Prevotellaceae*, involved in the formation of intestinal mucus, are associated with the severity of PD [114]. Several reports have suggested that *Prevotellaceae* are involved in mucin synthesis and ghrelin secretion and may play a protective role in the pathological process [114,116]. By contrast, *Akkermansia* may reduce barrier function and increase exposure to more microbial substrates across the epithelial lining. After transplantation into mice, microbiota from patients with PD induced gut inflammation, intestinal-barrier disruption, reduced CD4+ cell numbers, and increased circulating pro-inflammatory cytokines [117]. Furthermore, disease severity and changes in diet or lifestyle may also contribute to microbiome differences in PD [118,119]. Thus, gut dysbiosis may represent a cause, a consequence, or a modifier of PD-related pathological processes, and its precise role requires further longitudinal and interventional investigation.

### 4.2. Gut Infection Induces Increased Microbial Endotoxin and Brain Inflammation Through Blood

**Figure 2:**
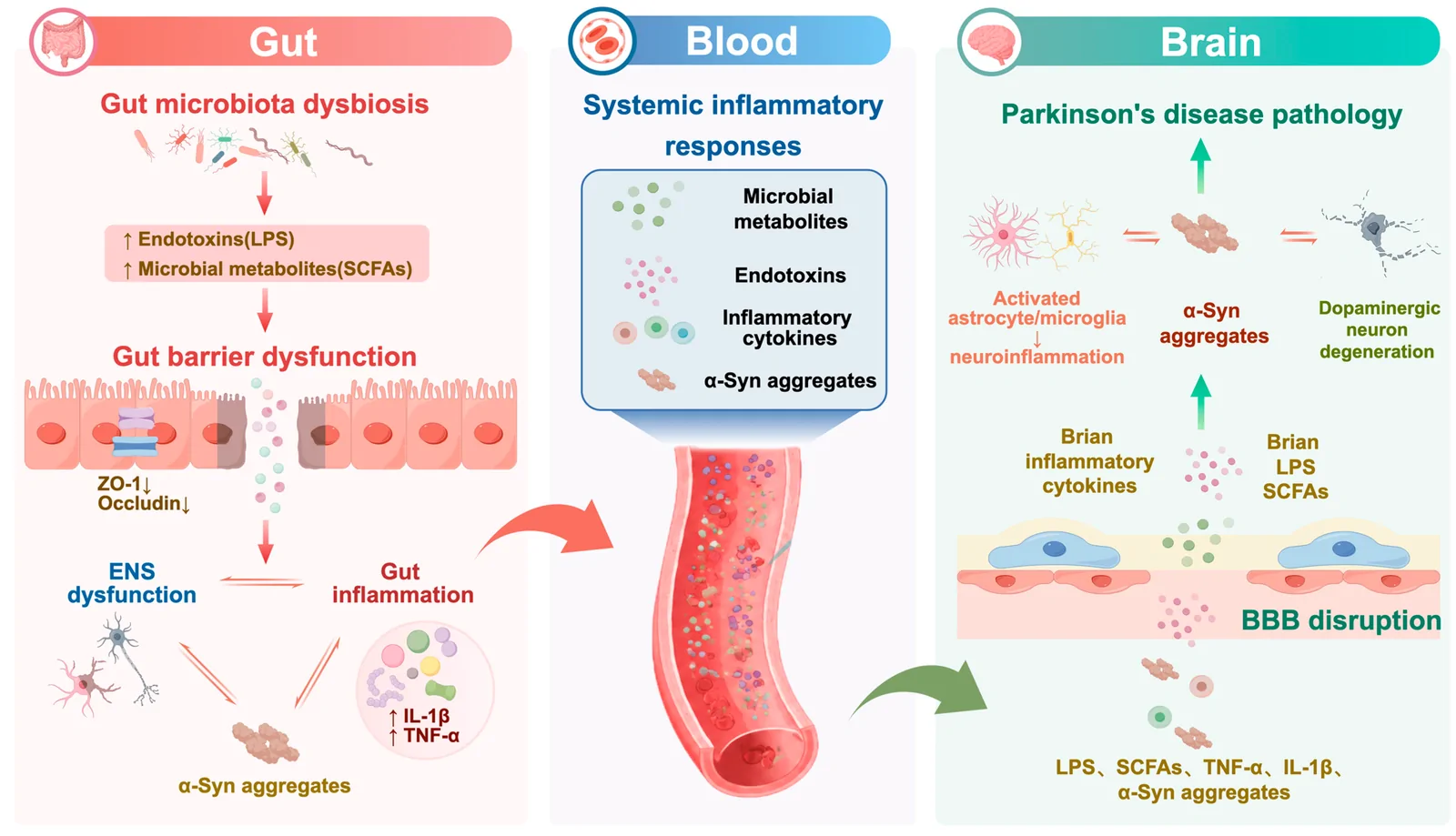
Gut microbiota dysbiosis-induced systemic inflammation in the blood and its effects on brain pathology in PD. Gut microbiota dysbiosis leads to elevated levels of endotoxins and microbial metabolites (e.g., short-chain fatty acids, SCFAs), which translocate across the compromised intestinal barrier into gut tissues. This process triggers local increases in pro-inflammatory cytokines (e.g., IL-1β, TNF-α) and subsequently induces ENS dysfunction and aberrant aggregation of α-Syn. The resulting α-Syn aggregates, inflammatory mediators, endotoxins, and metabolites enter systemic circulation, promoting systemic inflammatory responses. Under conditions of BBB disruption, these pathological factors infiltrate the brain, exacerbating accumulation of α-Syn, activation of astrocytes and microglia, and the degeneration of dopaminergic neurons. This figure was created by Figdraw.

Both central and peripheral inflammation may contribute to the pathogenesis and progression of PD [120,121]. Central neuroinflammation is characterized by the activation of microglia and astrocytes, together with T cell involvement. Peripheral inflammation involves activation of innate immune cells and T cell signaling in the ENS, the gastrointestinal tract, and the circulation [120,121]. Several findings have provided evidence that intestinal inflammation occurs in PD [6], and gastrointestinal mucosal damage is associated with an elevated risk of a subsequent clinical diagnosis of PD [122]. Intestinal macrophage activation may promote enteric α-Syn deposition. Circulating TNF-α and IL-1β levels are increased in some PD cohorts; under conditions of BBB disruption, peripheral inflammatory signals may influence central cytokine and chemokine expression [117]. Furthermore, blood cytokine levels have been associated with PD severity and progression [123] (Figure 2).

Intestinal infection or gut dysbiosis may increase endotoxin leakage into the circulation [124]. Epidemiological studies have associated certain bacterial, viral, and fungal infections with an increased risk of PD [125,126], but these associations do not establish causality. *H. pylori* have been proposed to influence systemic inflammation and PD-related outcomes; direct evidence that this process drives cytokine entry into the brain and dopaminergic-cell loss remains limited. Increased microbial burdens are positively correlated with pro-inflammatory cytokine levels. For instance, *H. pylori* infection may overwhelm the immune system, allowing immune-activated or inflammation-related molecules, such as IL-6 and TNF-α, to cross the BBB, leading to inflammation and degeneration of dopaminergic cells in the brain, ultimately contributing to PD [125]. PD patients often exhibit elevated plasma homocysteine levels, which may be influenced by *H. pylori* infection and in turn affect the progression of PD. In the colon, *T. gondii* infection is associated with intestinal dysbiosis, marked by heightened colonic inflammation, increased bacterial endotoxin translocation into the circulation, and compromised intestinal barrier function, further inducing anxiety-like behaviors in mice [127].

Elevated circulating endotoxin levels may impair BBB integrity and could thereby facilitate the entry of circulating pathological factors, including some α-Syn species. Endotoxin may cross the BBB bound to lipoproteins via lipoprotein transport mechanisms [128]. Elevated endotoxin levels in the brain then activate the innate immune system and microglia, provoking neuroinflammation and ultimately dopaminergic neuronal death [16]. Endotoxin has the potential to affect α-Syn conformation, with distinct LPS β-sheet structures driving alterations in fibril density and corresponding behavioral phenotypes in animal models [129].

LPS is a naturally occurring endotoxin produced by Gram-negative bacteria and is abundant in the gut [130]. Studies have shown that increased abundance of *Enterobacteriaceae* can lead to elevated serum LPS, with higher levels of LPS-binding proteins observed in the blood of PD patients, indicating increased absorption of LPS [131]. The intestinal epithelium blocks LPS entry; resident bacteria modulate barrier permeability by regulating tight-junction proteins [132,133]. Under physiological conditions, an intact intestinal barrier restricts the interaction of bacteria and LPS with epithelial cells. Disruption of the intestinal barrier allows bacteria and LPS to traverse the epithelium and enter the blood beneath the intestinal mucosa, thereby further impairing the intestinal epithelial barrier and triggering systemic inflammation via the TLR and NF-κB signaling pathways. This inflammatory response promotes the release of inflammatory cytokines, which in turn damage the BBB and facilitate α-Syn aggregation and deposition in the brain [91,131,134]. Systemic LPS administration in mice induced sustained brain TNF-α production and microglial activation, followed by delayed and progressive loss of nigral dopaminergic neurons that was more pronounced than the loss observed in adjacent ventral tegmental area neurons [135].

In addition to endotoxins, gut microorganisms may influence the absorption and bioavailability of dietary iron, which could affect systemic iron homeostasis [136]. For example, Lactobacillus fermentum and its metabolites can enhance iron absorption and uptake by ECs [137]. Individuals with hereditary peripheral iron overload have an increased risk of idiopathic PD. Inflammatory bowel disease (IBD) has been epidemiologically associated with an increased risk of PD and is characterized by toxic accumulation of iron in the gut [136]. As iron absorption increases, the iron concentration in the blood may change. Evidence from meta-analyses indicates that PD patients exhibit significantly higher serum iron levels than healthy controls [138]. Increased BBB permeability or dysfunction may promote iron deposition in the brain, including in the substantia nigra pars compacta and reticulata, and is associated with PD severity [139,140]. This iron overload can modify microglial phenotypes, promote the secretion of inflammatory cytokines following brain inflammation, and increase neurotoxicity [141].

### 4.3. Gut Microbiome-Driven Changes in Circulating Metabolites and Their Effects on the Brain

Gut microbiota-derived metabolites can cross the intestinal epithelial barrier and enter the circulation, where they may act as signaling molecules that influence synucleinopathy-related processes. Compared with healthy controls, patients with PD have shown altered plasma concentrations of several metabolites, including short-chain fatty acids (SCFAs), indole-3-propionic acid (IPA), deoxycholic acid, and glycodeoxycholic acid [142,143]. One study identified six circulating metabolites associated with gut microbial alterations and proposed candidate microbial species that might contribute to these metabolic differences. These gut-driven changed metabolites may further spread into the brain through the blood and influence neurodegeneration. For instance, lipoteichoic acid from *Bacillus subtilis* increased plasma levels of pro-inflammatory cytokines and induced BBB dysfunction, contributing to peripheral and brain inflammation [144].

#### 4.3.1. Short-Chain Fatty Acids (SCFAs)

SCFAs, a class of organic fatty acids produced by the gut microbiota, such as acetate, propionate, and butyrate, contribute to the maintenance of BBB integrity. SCFAs interact with intestinal epithelial cells to regulate immune and inflammatory responses both locally in the gut and systemically, enhancing gut barrier function. However, SCFA activity is not restricted to the intestine. A small proportion enters the systemic circulation after absorption across the colonic epithelium and can cross the BBB via transporters on brain endothelial cells [145]. In experimental studies, SCFAs can suppress microglial cytokine release and enhance endothelial function [146].

Fecal SCFA levels were reduced in PD, whereas plasma levels were increased. Higher plasma SCFA levels were associated with altered GVB permeability and constipation [142]. SCFAs can also promote tight-junction protein expression through G protein-coupled receptors, thereby enhancing BBB integrity [147]. Some studies have indicated that SCFAs may have adverse effects in PD. Low levels of SCFAs are detectable in the human brain, where they modulate neurotransmitters such as glutamate, glutamine, GABA, and neurotrophic factors [148]. In particular, acetate has been shown to inhibit the expression of epithelial cell-related proteins, such as ZO-1 and occludin, leading to intestinal inflammation, impairment of the gut barrier, and altered BBB integrity [149]. Reducing acetate levels can help suppress the expression of pro-inflammatory factors, whereas butyrate supplementation can reduce neuronal damage by increasing the expression of occludin and ZO-1, both of which contribute to restoring BBB integrity [149]. Butyrate may improve barrier function by increasing MUC2 expression and activating AMPK-related pathways; improved barrier function can be reflected by increased transepithelial electrical resistance (TEER) [150].

#### 4.3.2. Bile Acids (BAs)

Primary BAs are produced by hepatocytes and stored in the gallbladder, whereas secondary BAs are metabolized by intestinal bacteria, such as *Bacteroides*, *Bifidobacterium*, *Clostridium*, *Lactobacillus*, and *Listeria*, among others, which are dysregulated in the PD gut [151]. LC-MS analysis of clinical plasma samples showed alterations in two bile acids (3β–Hydroxy-5-cholenoic acid and glycoursodeoxycholic acid) in patients with PD [152]. After aerobic exercise, bile acid metabolic profiling in PD patients indicated a significant reduction in fecal 7-ketolithocholic acid concentration and decreases in serum taurochenodeoxycholic acid and taurodeoxycholic acid [153]. Ursodeoxycholic acid (UDCA) and its taurine conjugate (TUDCA) were decreased in the plasma of PD patients, although the difference was not statistically significant [154]. These compounds have been shown to cross the BBB and exert neuroprotective effects, including reducing neuroinflammation, alleviating mitochondrial dysfunction, and inhibiting proteins involved in apoptosis [155]. Furthermore, treatment with UDCA or TUDCA could improve motor performance, inhibit mitochondrial dysfunction and neuroinflammation, and prevent the decline in striatal dopamine content [156,157].

#### 4.3.3. Amino Acids

Amino acids are essential for protein and peptide synthesis, which is linked to the activity of multiple bacterial species, such as *Klebsiella* spp., *Escherichia coli*, and *Anaerovibrio lipolytica* [158]. These bacteria contribute to amino-acid production and utilization. Amino acids can be absorbed from the gut into the circulation, although their causal contribution to PD remains uncertain. Plasma profiling of BCAAs and AAAs has revealed significantly lower levels in PD [159].

IPA has been shown to increase transepithelial electrical resistance and decrease paracellular permeability in the intestinal tract [160,161]. Consistent with its effects on gut barrier integrity, IPA has been shown to maintain BBB integrity, improve endothelial function, and exert protective effects via activation of FFAR3 [162]. Moreover, given its capacity to readily enter the central compartment, IPA may exert direct effects on brain function and cell survival [163]. In cellular models of PD, IPA reduced endoplasmic reticulum stress, oxidative stress, and inflammatory gene expression [164]. Plasma IPA levels are increased in PD patients relative to age- and sex-matched controls and show no correlation with cognitive or motor performance but are associated with brain-derived neurotrophic factor levels [143,165]. The elevated plasma levels of IPA may reflect a compensatory response in PD.

#### 4.3.4. Trimethylamine N-Oxide (TMAO)

Gut bacteria can produce trimethylamine (TMA) from dietary precursors; TMA is then absorbed and oxidized to TMAO by hepatic flavin-containing monooxygenases. As a gut microbial metabolite capable of crossing the BBB, direct dietary intake of TMAO can exacerbate multiple key pathological alterations in PD model mice [166]. TMAO may also impair the cellular functions of the brain glymphatic system and meningeal lymphatic vessels, and enhance intracranial inflammation [167]. *E. fergusonii* serves as an important source of TMAO production, and its abnormal enrichment in PD contributes to increased TMAO levels and altered inflammatory responses, which can be ameliorated by F.l-HP combination therapy [166]. An increasing number of studies have demonstrated higher plasma TMAO concentrations in patients with PD, which are associated with both disease severity and motor symptoms [168,169]. In α-Syn overexpressing mice, TMAO levels were strongly correlated between the gastrointestinal tract and plasma, as well as between plasma and brain tissues [170]. Furthermore, TMAO has been reported to promote α-Syn fibrillization and aggregation in vitro [170].

## 5. Application of the Gut–Blood–Brain Axis

### 5.1. Blood Biomarkers Related to Gastrointestinal Dysfunction and Brain Pathology in PD

Recent studies have provided increasing evidence for a potential link between gut health and PD, with blood biomarkers playing a significant role in this association. Changes in the levels of certain blood biomarkers, such as α-Syn, cytokines, metabolites, and microbiome-related substances, may reflect the impact of gut microbiota dysbiosis in PD (Table 2). Although no correlation was observed between the levels of inflammatory markers in plasma and feces from the same PD patients, the study revealed that inflammation-associated molecules were highly correlated within each sample type [171]. Elevated fecal IL-8 levels were associated with increased constipation severity in PD, and both IL-8 and IL-1β were also increased in an independent cohort of PD patients [172]. Similar inflammatory changes have been reported in plasma from patients with PD, and serum IL-8 levels have been positively associated with PD-related disability [173,174].

Several markers of intestinal inflammation and permeability, including β-defensin 2, zonulin, and lactoferrin, were increased in stool samples from patients with PD [182]. Plasma lactoferrin did not differ between PD and control groups but was inversely associated with PD severity [186]. Both zonulin and calprotectin were higher in serum from PD patients [178]. Fatty acid-binding protein (FABP) is a lipid chaperone that transports lipids into specific compartments and has subtypes with different localization patterns [187]. Analysis of plasma protein levels in PD patients revealed that plasma FABP2 levels fluctuated with disease duration and showed a significant negative correlation with α-Syn levels [188]. Furthermore, fluctuations in the FABP2/α-Syn ratio demonstrated greater accuracy in differentiating disease groups from healthy controls than using FABP2 or α-Syn levels alone [188].

The levels of metabolites were significantly increased in the plasma of PD patients relative to healthy controls, whereas no significant differences were detected in fecal samples between the groups [189]. SCFAs are found at low concentrations in feces, whereas their plasma levels are elevated, potentially due to impaired gut barrier function [142,143]. However, correlation analysis revealed that elevated levels of differential metabolites in the plasma of PD patients were associated with increased intestinal permeability and inflammation [189]. Functional prediction of gut microbiota suggested altered BCAA metabolism, which resulted in the accumulation of BCAAs in mouse colonic organoids and peripheral blood, as well as in serum from PD patients [190].

Both PD and IBD exhibit similar peripheral blood alterations, including chronic inflammation, iron dysregulation, and mitochondrial dysfunction in immune cells, suggesting potential shared pathogenic mechanisms. These common hematological features may serve as valuable biomarkers for early diagnosis and monitoring disease progression in both conditions, particularly given their epidemiological association [136]. For example, neutrophil gelatinase-associated lipocalin (NGAL), an antimicrobial glycoprotein originating from epithelial cells and highly expressed under inflammatory conditions, has been identified as a potential diagnostic and disease activity biomarker for IBD and is also increased in the plasma of PD patients compared with controls [173,191].

### 5.2. Therapeutic Strategies Targeting the Gut–Brain Axis

Oral pharmacotherapy remains central to the symptomatic management of PD, and the gut microbiota can influence drug bioavailability. A high abundance of gut bacterial tyrosine decarboxylase influences levodopa absorption in the proximal small intestine, which has a substantial effect on plasma levodopa levels in PD rats [192]. In experimental models, microbiota depletion or inhibition of bacterial levodopa metabolism increased levodopa availability [193,194,195]. Additionally, administration of Lactobacillus has been shown to attenuate BBB and intestinal barrier permeability in a 6-OHDA PD model by reducing oxidative stress [196]. Dietary intervention with a combination of two precursors of membrane synthesis, uridine and docosahexaenoic acid, can protect against neurodegeneration and improve motor and non-motor symptoms in a rotenone model of PD [197].

Specific differences in fecal samples from PD patients compared with those from healthy controls have been identified, such as an association between *Bacteroides* abundance and inflammatory markers in the blood and motor deficits [198]. These findings support continued investigation of microbiota-targeted therapeutic strategies. Fecal microbiota transplantation has been shown to correct gut microbiota dysbiosis and decrease LPS levels in the colon, serum, and SN, leading to suppression of the TLR4/MyD88/NF-κB signaling cascade and its associated pro-inflammatory mediators [199]. Rifaximin reduced serum IL-1β, IL-6, and TNF-α and increased or preserved claudin-5 and occludin expression, consistent with reduced systemic inflammation and improved gut-barrier and BBB integrity [200].

Traditional Chinese medicine and acupuncture have also been investigated as modulators of the gut–brain axis and circulating inflammatory mediators. Preclinical studies suggest that acupuncture may ameliorate dysbiosis, suppress systemic inflammation, and reduce α-Syn expression [201,202]. However, clinical efficacy and mechanisms require further confirmation. After acupuncture treatment at the GB34 and ST36 acupoints, the gut microbiota composition was partially restored in MPTP-treated mice. Changes in bacterial genera, including *Butyricimonas* and *Holdemania*, were positively correlated with motor function and were associated with changes in circulating butyrate levels, which may contribute to the anti-inflammatory effects of acupuncture [203].

## 6. Conclusions

PD is a multisystem disorder in which the gut–brain axis may contribute to disease progression through multiple interacting mechanisms. Although it has been hypothesized that α-Syn propagates via the ENS–vagal–spinal pathway, this mechanism alone cannot fully explain PD pathology. As an anatomically continuous circulatory network connecting organs and tissues throughout the body, the peripheral blood circulation may serve as an additional medium between the gut and the brain, raising the possibility that it contributes to communication within the gut–brain axis. Experimental studies support the plausibility of this route. Different α-Syn species, gut-derived microbial products and metabolites, and peripheral inflammatory mediators can interact with the intestinal vascular barrier and BBB and thereby influence peripheral and central pathology.

However, current evidence is more consistent with the gut–blood–brain axis modifying pathology in some patients than with its causing PD. Most human evidence remains observational and cannot exclude reverse causality or confounding. Gastrointestinal dysmotility, constipation, dietary changes, and medication use may themselves contribute to the microbiota alterations observed in PD. Direct evidence demonstrating a complete gut-to-blood-to-brain causal pathway in human PD remains limited. Longitudinal human cohorts with paired sampling of gut, blood, cerebrospinal fluid, and brain tissue are needed, as are molecular tracing studies to determine where pathological species originate and interventions that block the blood-mediated route. Clarifying these mechanisms will be essential for determining whether biomarkers and therapeutic strategies targeting the gut–blood–brain axis have clinical value.

## Figures and Tables

**Table 1:** Changes in pathological α-Syn in the gut, blood and brain in PD.

Different Forms α-Syn	Gut			Blood			Brain		
	Sample	Changes	Methods	Sample	Changes	Methods	Sample	Changes	Methods
Total α-Syn	Stomach	Up↑ [38]	Immunohistochemical analysis	Serum	Up↑ [39]	Immunomagnetic reduction (IMR)	Substantia nigra	Up↑ [7]	Immunohistochemical analysis
	Gastrointestinal tissues	Unchanged [40]	Immunohistochemical analysis	Plasma	Up↑ [34]	Enzyme linked immunosorbent assay (ELISA)	Cortex	Up↑ [7]	Immunohistochemical analysis
	Colon	Up↑ [41]	Immunohistochemical analysis	Plasma	Down↓ [33]	Western blot analysis	CSF	Down↓ [42]	ELISA
	Colon	Unchanged [43,44]	Immunohistochemical analysis	RBCs	Up↑ [45]	Electrochemilumin-escence (ECL) immunoassay	CSF	Unchanged [46]	ELISA
				RBCs cytosolic α-Syn	Unchanged [47]	ECL immunoassay			
				RBCs membrane α-Syn	Up↑ [47]	ECL immunoassay			
α-Syn aggregates	Stomach	Up↑ [48]	Immunohistochemical analysis	Serum	Up↑ [49]	ELISA	Substantia nigra	Up↑ [50]	Immuno-based assays
	Gastro- Intestinal Biopsy tissue	Unchanged [51]	Proximity ligation assay (PLA)	Plasma	Up↑ [52]	ELISA	Cortex	Up↑ [53]	Paraffin-embedded tissue blot
				Plasma	Down↓ [54]	ELISA	CSF	Up↑ [55]	ELISA
				RBCs	Up↑ [37]	ELISA			
Phosphorylated α-Syn	Colon	Up↑ [56]	Immunohistochemical analysis	Serum	Unchanged [57]	ELISA	Substantia nigra and cortex	Up↑ [50]	ELISA
	Colon	Up↑ [41]	Immunohistochemical analysis	Plasma	Up↑ [58]	ELISA	Frontal cortex	Down↓ [50]	Mass spectrometry
				Plasma	Unchanged [59]	ELISA	CSF	Up↑ [60]	ELISA
	Small intestine and colon	Down↓ [61]	Immunohistochemical techniques	RBCs	Up↑ [47]	ECL immunoassay	CSF	Down↓ [62]	SIMOA
	Stomach and colon	Unchanged [63]	Immunohistochemical analysis		Up↑ [35]	ELISA	CSF	Unchanged [64]	ECL immunoassay
EV- associated α-Syn	/	/	/	Serum	Down↓ [65]	ELISA	CSF	Down↓ [66]	ECL immunoassay
				Plasma	Up↑ [67]	ECL immunoassay	CSF	Down↓ [68]	Apogee nanoscale flow cytometry technology
α-Syn fibril (seed activity)	Gastric biopsies	Up↑ [69]	Real-time quaking-induced conversion (RT-QuIC)	Serum	Up↑ [70]	Nanoparticle-enhanced Quaking- induced Conversion (Nano-QuIC)	Substantia nigra	Up↑ [71]	RT-QuIC
	Duodenum	Up↑ [22]	RT-QuIC	Plasma	Up↑ [70]	Nano-QuIC	CSF	Up↑ [72]	RT-QuIC
	Colon	Up↑ [73]	RT-QuIC				CSF	Up↑ [72]	Protein misfolding cyclic amplification (PMCA)

**Table 2:** Blood biomarkers related to gastrointestinal disruptions in PD.

Type	Biomarker	Changes in Blood		Changes in Gut	
		Blood Fraction	Changes	Tissue or Sample	Changes
Inflammatory cytokines	IL-1β	Plasma	Unchanged [116,175]	Colon	Up↑ [176]
		Blood	Up↑ [177]		
	TNF-α	Plasma	Up↑ [116,175]	Feces	Up↑ [175]
	IFN-γ	Plasma	Up↑ [116]	Colon	Up↑ [176]
		Blood	Down↓ [177]		
	IL-6	Serum Plasma	Up↑ [58,123]	Feces	Up↑ [171]
	IL-8	Serum Plasma	Up↑ [173,174]	Feces	Up↑ [171,172]
Intestinal permeability marker	Zonulin	Serum	Up↑ [178,179]	Stomach and jejunum	Up↑ [180]
				Feces	Unchanged [181]
				Feces	Up↑ [182]
Microbial metabolites	Short chain Fatty acids (SCFAs)	Plasma	Up↑ [142]	Feces	Down↓ [142]
	Bile acids (BAs)	Plasma	Up↑ [143]	Ileum	Up↑ [183]
			Down↓ [154,155]		
	Indole -3- propionic acid (IPA)	Plasma	Up↑ [143,165]	Colon	Up↑ [184]
	Trimethylamine N-oxide (TMAO)	Plasma	Up↑ [168,169]	Feces	Up↑ [185]
Others	Calprotectin	Serum	Up↑ [178]	Feces	Up↑ [181]
	Lactoferrin	Plasma	Unchanged [186]	Feces	Up↑ [182]
	Neutrophil gelatinase-associated lipocalin (NGAL)	Plasma	Up↑ [173]	Feces	Up↑ [171]

## Data Availability

No new data were created or analyzed in this study. Data sharing is not applicable to this article.

## Abbreviations

The following abbreviations are used in this manuscript:

AAAS	Aromatic Amino Acids
α-Syn	α-Synuclein
BAs	Bile Acids
BCAAs	Branched-Chain Amino Acids
BBB	Blood–Brain Barrier
CNS	Central Nervous System
CSF	Cerebrospinal Fluid
DA	Dopamine
ECs	Endothelial Cells
EECs	Enteroendocrine Cells
ENS	Enteric Nervous System
EVs	Extracellular Vesicles
FABP	Fatty Acid-Binding Protein
FABP2	Intestinal-Type Fatty Acid-Binding Protein
FFAR3	Free Fatty Acid Receptor 3
GI	Gastrointestinal
GIT	Gastrointestinal Tract
GVB	Gut–Vascular Barrier
IBD	Inflammatory Bowel Disease
IFN-β	Interferon Beta
IFN-γ	Interferon Gamma
IL-1β	Interleukin-1 Beta
IL-6	Interleukin-6
IL-8	Interleukin-8
IL-13Rα1	Interleukin-13 Receptor Alpha 1
IPA	Indole-3-Propionic Acid
LAG3	Lymphocyte-Activation Gene 3
LBs	Lewy Bodies
LPS	Lipopolysaccharide
LRP-1	Low-Density Lipoprotein Receptor-Related Protein 1
MPTP	1-methyl-4-phenyl-1,2,3,6-tetrahydropyridine
MSC	Mesenchymal Stem Cell
NF-κB	Nuclear Factor κB
NGAL	Neutrophil Gelatinase-Associated Lipocalin
NO	Nitric Oxide
PBMCs	Peripheral Blood Mononuclear Cells
PD	Parkinson’s Disease
RBCs	Red Blood Cells
ROS	Reactive Oxygen Species
RT-QuIC	Real-Time Quaking-Induced Conversion
SCFAs	Short-Chain Fatty Acids
SN	Substantia Nigra
TEER	Transepithelial Electrical Resistance
TLR4	Toll-Like Receptor 4
TMAO	Trimethylamine N-Oxide
TUDCA	Tauroursodeoxycholic Acid
UDCA	Ursodeoxycholic Acid
VEGFA	Vascular Endothelial Growth Factor A
ZO-1	Zonula Occludens-1
